# Subconjunctival fat prolapse: a disease little known to radiologists

**DOI:** 10.1590/0100-3984.2015.0229

**Published:** 2017

**Authors:** Cynthia Ramos Tejo, Péricles Almeida da Costa, Rafaella Martins Batista, Yuri Raoni Ramalho Rocha, Marcelle Alves Borba

**Affiliations:** 1 Universidade Federal do Rio Grande do Norte (UFRN), Natal, RN, Brazil.

**Figure 1:**
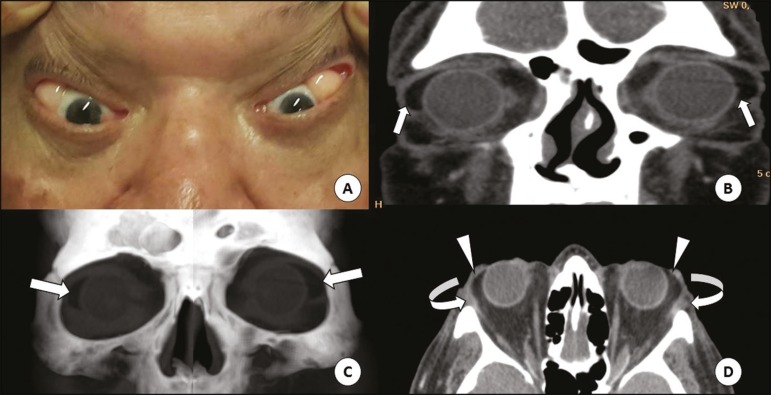
**A:** Fatty mass in the lateral corner of the orbits, best characterized by the retropulsion of the globes. CT of the orbits in the coronal plane (**B**), with volumetric reconstructions in the coronal plane (**C**) and axial plane (**D**), showing the masses in the lateral corners (straight arrows), contiguous with the intraconal fat (arrowheads) and pushing aside the lacrimal glands (curved arrows).

Dear Editor,

A 69-year-old male patient sought outpatient treatment with a 10-year history of fatty masses in the lateral corners of his eyes, best characterized as retropulsion of the globes. He underwent computed tomography (CT) of the orbits, which revealed intraconal fat proliferation in the lateral corners of the eyes, from the orbits to the epibulbar region (Figure 1). Given the clinical presentation and imaging findings, a diagnosis of subconjunctival fat prolapse was made.

Subconjunctival fat prolapse is an acquired, typically bilateral, condition characterized by herniation of the intraconal fat, resulting from weakness of the eyeball and intermuscular septum due to aging, trauma, or surgery^(1)^. It is more common in obese men between the 7th and 8th decades of life and manifests clinically as a yellowish mass in the lateral corner of the eye, which becomes more evident with retropulsion of the globe^(2)^.

The imaging tests that can facilitate the diagnosis of subconjunctival fat prolapse are CT and magnetic resonance imaging (MRI) of the orbits, the most important radiological finding being that of a mass with fat density or fat-like signal intensity, respectively, located in the temporal aspect of the orbits, contiguous with intraconal fat.

The treatment consists of transconjunctival excision, a simple, safe and effective surgical procedure. The rate of recurrence after transconjunctival excision is reported to be approximately 9%^(3)^.

Making a clinical diagnosis of subconjunctival fat prolapse is relatively easy. However, due to its rarity, it can be misdiagnosed as conjunctival dermolipoma, lymphoma, epidermoid cyst, or lacrimal gland prolapse^(4)^. The main differential diagnosis is conjunctival dermolipoma, which consists of a benign lesion, usually present at birth^(5)^, that affects young women, the mean age of such patients being 22 years^(6)^. Although the clinical presentation of conjunctival dermolipoma is similar to that of the subconjunctival fat prolapse, the former is typically unilateral and fairly immobile. On CT and MRI, conjunctival dermolipoma presents as a crescent-shaped fatty mass in the temporal aspect of the orbit, not in communication with the intraconal fat^(1)^.

In conjunctival dermolipoma, surgical resection is indicated mainly for aesthetic purposes and tends to be more conservative^(1)^. Although resection of a conjunctival dermolipoma is a simple procedure, there can be severe complications, including blepharoptosis, diplopia, and keratoconjunctivitis sicca. Therefore, a number of different surgical techniques aimed at a lowering the rate of complications and improving the aesthetic results have been described, including resection with conjunctival flap rotation^(7)^.

Subconjunctival fat prolapse and dermolipoma present clinically as a fatty epibulbar masses in the lateral corners of the orbits, and in some cases their differentiation by clinical aspects can be difficult. The subject is little known among radiologists, and there have been few reports of related cases. Therefore, given the difference between these two entities in terms of treatment, it is necessary that radiologists be familiar with both, in order to recognize them promptly and make the differential diagnosis through the use of imaging tests.
